# A Review of Dietary Surveys in the Adult South African Population from 2000 to 2015

**DOI:** 10.3390/nu7095389

**Published:** 2015-09-23

**Authors:** Zandile J. Mchiza, Nelia P. Steyn, Jillian Hill, Annamarie Kruger, Hettie Schönfeldt, Johanna Nel, Edelweiss Wentzel-Viljoen

**Affiliations:** 1 Population Health, Health Systems and Innovation of the Human Sciences Research Council (PHHSI) of South Africa, 12th Floor, Plein Park Building, 69–83 Plein Street, Cape Town 8001, South Africa; 2 Division of Human Nutrition, University of Cape Town (UCT), UCT Medical Campus, Anzio Road, Observatory, Cape Town 7925, South Africa; E-Mail: nelia.steyn@uct.ac.za; 3 Non-Communicable Diseases Research Unit, Medical Research Council (NCDRU) of South Africa, Francie van Zijl Drive, Parrow Valley, Cape Town 7505, South Africa; E-Mail: jillian.hill@mrc.ac.za; 4 Africa Unit for Transdisciplinary Health Research (AUTHeR), Faculty of Health Sciences, North-West University, Potchefstroom 2520, South Africa; E-Mail: Annamarie.Kruger@nwu.ac.za; 5 Medical Research Council Research Unit for Hypertension and Cardiovascular Disease, Faculty of Health Sciences, North-West University, Potchefstroom 2520, South Africa; E-Mail: edelweiss.wentzel-viljoen@nwu.ac.za; 6 Department of Animal and Wildlife Sciences, University of Pretoria, Hatfield, Pretoria 0028, South Africa; E-Mail: Hettie.schonfeldt@up.ac.za; 7 Department of Logistics, University of Stellenbosch, Matieland, Stellenbosch 7602, South Africa; E-Mail: jhnel@sun.ac.za; 8 Centre for Excellence in Nutrition (CEN), Faculty of Health Sciences, North-West University, Potchefstroom 2520, South Africa

**Keywords:** macronutrients, micronutrients, food consumption, dietary diversity, South Africans, food intake

## Abstract

One serious concern of health policymakers in South Africa is the fact that there is no national data on the dietary intake of adult South Africans. The only national dietary study was done in children in 1999. Hence, it becomes difficult to plan intervention and strategies to combat malnutrition without national data on adults. The current review consequently assessed all dietary studies in adults from 2000 to June 2015 in an attempt to portray typical adult dietary intakes and to assess possible dietary deficiencies. Notable findings were that, in South Africa micronutrient deficiencies are still highly prevalent and energy intakes varied between very low intakes in informal settlements to very high intakes in urban centers. The most commonly deficient food groups observed are fruit and vegetables, and dairy. This has been attributed to high prices and lack of availability of these food groups in poorer urban areas and townships. In rural areas, access to healthy foods also remains a problem. A national nutrition monitoring system is recommended in order to identify dietary deficiencies in specific population groups.

## 1. Introduction

There is a dearth of national data regarding the dietary intake of adult South Africans since there has never been a national study on adults. The only national survey to date was in children one to nine years old in 1999 (National Food Consumption Survey, NFCS) [1]. As a result, local, isolated and fragmented dietary intake studies have been used by nutrition professionals and decision-makers in an effort to understand the nutrient intake of adult South Africans. 

These studies included, among others: Coronary Risk Factor Study (CORIS and CRISIC) [2,3,4]; Black Risk Factors Study (BRISK) [5,6]; Weight and Risk Factor Study (WRFS) [7]; Dikgale Study [8]; Transition, Health and Urbanization Study (THUSA) [9,10,11] and First Year Women Students (FYWS) [12,13]. Furthermore, in 2002, secondary dietary analyses of the data obtained from these studies was undertaken on dietary intake data of adults published prior to 2000 and with the aid of modeling data of larger studies, an average daily intake was generated for adults in South Africa [14,15]. The data generated by secondary data analyses indicated that certain nutrients were deficient in the diet of some individuals of the adult population. These included: calcium, iron, zinc, riboflavin, niacin, folate, and vitamins B6, A, E and C [16]. In addition to the nutrient intakes, data of commonly consumed food items and their portion sizes was also generated. 

Fifteen years have passed since the secondary data analyses took place and there still has not been a national dietary study on adults. It is regarded as being important to repeat the process undertaken in 2002 and to assess dietary studies undertaken after 2000 to date and to extrapolate data on macro- and micronutrients, foods consumed and dietary diversity. This data will allow for more accurate planning of targeted interventions to curb serious under- and over-nutrition and nutrient deficiencies by nutrition professionals and policy makers at local and national level.

## 2. Methods

The methods used were in accordance with the methods described for doing a systematic review, namely: framing the research question; identifying relevant work; analyzing the quality of studies; summarizing the evidence; and interpreting the findings [17].

### 2.1. Framing the Research Question

The aim of this review was: (i) to identify dietary studies on adults which took place in South Africa after 2000 and to categorize the data according to gender, age and geographic location, including rural and urban areas; and (ii) to summarize the data in a manner which would provide valuable insight into possible nutrient deficiencies and commonly eaten foods. 

### 2.2. Identifying Relevant Work

This review employed electronic and manual searching of peer reviewed literature, as well as electronic data sets of unpublished studies done on the dietary intake of adults since 2000. 

The databases searched were MEDLINE via EBSCOHOST, PubMed and ScienceDirect. For MEDLINE and PubMed, we used the following keywords: “dietary intake” OR “energy intake” OR “food intake” OR “food habits” OR “diet surveys” AND “South Africa” OR “South African” since 2000 and all in title/abstract and humans only. These terms were selected in accordance with the National Library of Medicine’s Medical Subject Headings. In addition, ScienceDirect was searched using the terms: “dietary intake” (in title and abstract) AND “South Africa” and limited to 2000 to date. Furthermore, the South African Journal of Clinical Nutrition was hand-searched from the year 2000 for dietary studies on adults. Specific Departments of Nutrition/Dietetics in South Africa were also contacted in order not to miss unpublished studies. 

Studies were included in the review according to the following inclusion criteria:

- Dietary studies which used one of the following methods: 24 h recall, food frequency, weighed dietary record, dietary history to record adult intakes.
- Participants in the studies were at least 14 years old.
- Studies included in the review had at least 30 participants per group.
- The study results included macro/micro nutrient intakes or foods commonly consumed or dietary diversity data or measures of dietary inadequacy.

Studies were excluded for the following reasons:

- Participants were breastfeeding or pregnant.
- Participants of the studies had a specific disease condition, e.g., diabetes or AIDS.
- Participants were disabled.

### 2.3. Analyzing the Quality of Studies

All the studies were read by the first three authors and agreement was reached regarding the inclusion and quality of the studies. Almost all studies used standardized (age- and culture-specific and previously validated) questionnaires to measure the dietary intake of participants. To be specific, five studies used a quantified 24-h recall (recalling all the food and drinks consumed the previous day), three used an unquantified 24-h recall (to measure food diversity, which is determined by counting food groups consumed) and the rest (*n* = 6) used a quantified food frequency questionnaire to measure dietary intake over a specific period, usually one month. The South African Medical Research Council (SAMRC) FoodFinder database [18] was used in all studies to analyze the dietary data collected with the exception of Kolahdooz *et al.* [19] who adopted the United States version (Nutribase) database to analyze and determine the nutrient content of food consumed by the participants. Because of the paucity of data in KwaZulu Natal province, Kolahdooz *et al.* [19] was included.

## 3. Summarizing the Evidence

**Figure 1:**
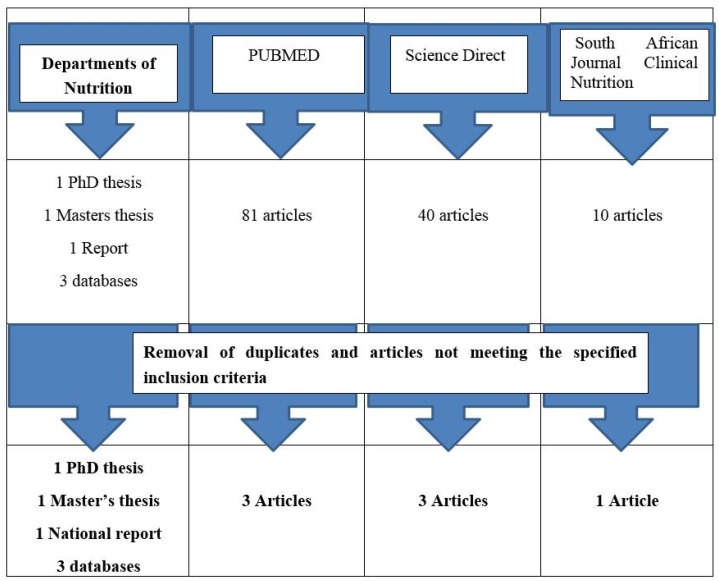
Schematic presentation of the literature search undertaken to find dietary surveys done in South Africa after 2000.

Results of the PubMed search yielded 81 studies, ScienceDirect yielded 40 studies, the South African Journal of Clinical Nutrition yielded 10 studies and contacts to Departments of Nutrition/Dietetics in South Africa produced one PhD thesis, one Master’s thesis, one report and three raw datasets. After removal of studies not meeting the criteria and duplicates, we were left with seven peer-reviewed studies, one PhD thesis, one Master’s thesis, and three raw databases; a total of 13 studies (Figure 1). These databases are the unpublished dietary data of three studies, namely: the prospective urban and rural epidemiological (PURE) study designed to track the changing lifestyles, risk factors and chronic disease among the South African population in 2005 and 2010 in the North West province [20,21]; and the cardiovascular risk in black South Africans (CRIBSA) study designed to measure the dietary intake of the urban black population in Cape Town in 2009 [22]. Almost all the studies used in this review are on the African population, with the exception of one study on the urban Indian population [23] and those which measured dietary variety. Data from these reviewed studies and databases are classified into four sections: macronutrient intakes, micronutrient intakes, foods consumed, and dietary diversity. Where possible, we have compared the intakes with the dietary reference intakes (DRIs) [24], usually the estimated average requirements (EARs), adequate intakes (AIs), recommended dietary allowances (RDAs) and acceptable macronutrient distribution range (AMDR).

Data on dietary intake namely: macronutrients, food eaten, and dietary diversity score are expressed as means and standard deviations. Furthermore, the minimum and maximum mean intakes of men and women for every micronutrient in all the presented studies were noted. As such, the data is reported as the lowest minimum value and highest maximum value for each nutrient reported to provide a range from lowest to highest for each micronutrient for the studies included. 

Table 1 presents data based on 13 studies that investigated the dietary intake of South Africans at local level (*i.e.*, within South African provinces). For comparison purposes, the secondary dietary analysis undertaken on studies before 2000 has been included in the table [14,15]. The data includes information that is gender-, age-, ethnic-, and local-specific. Altogether, sample sizes of the studies varied between 136 and 13,357. In addition, Table 2 presents the instruments and methodology used in the studies. Table 2 presents the studies reviewed with regards to the aims and methods used. 

### 3.1. Macronutrient Intakes

**Figure 2:**
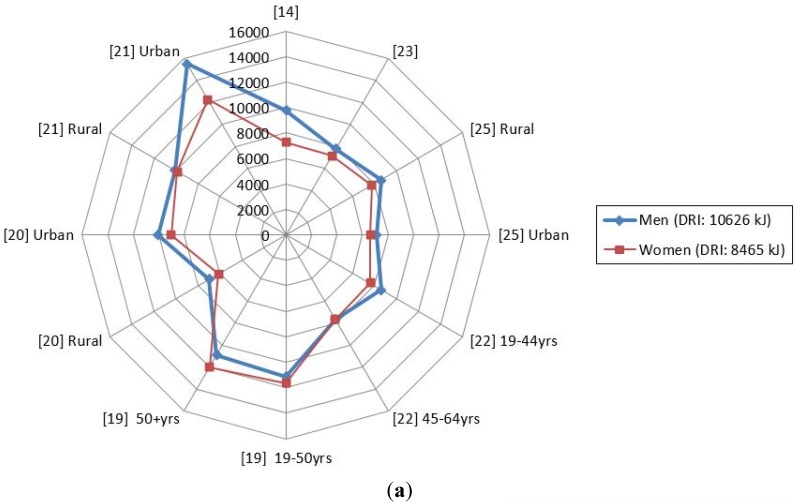

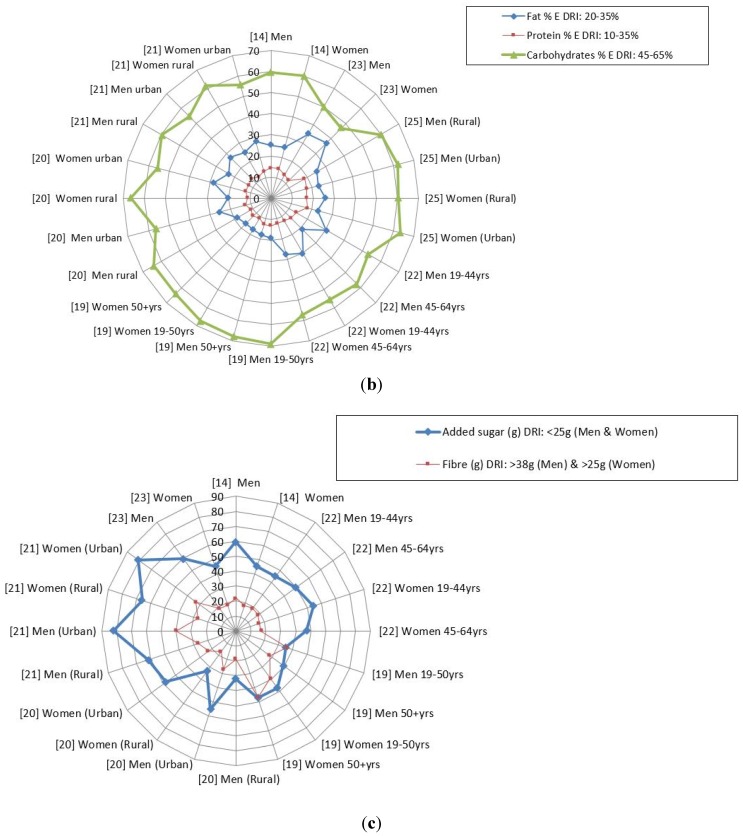
(**a**) Mean total energy intake (kilo Joules, kJ) consumed by South African men and women based on the studies undertaken after 2000; (**b**) Mean percentage contribution of macronutrients to the total energy intake of South Africans based on the studies undertaken after 2000; (**c**) Mean added sugar (g) and fiber (g) intake of South Africans based on the studies undertaken after 2000. Nel and Steyn [14]; Kolahdooz *et al.* [19]; Wentzel-Viljoen and Kruger [20,21]; Jaffer *et al*. [22]; Naicker [23]; Tydeman-Edwards [26].

According to Table 3 and Figure 2a, the total mean energy intakes of South Africans appear to lie below the DRIs for both men and women with the exception of energy intake of women in KwaZulu Natal [19] and urban men and women in the North West province [21]. In terms of the percent of energy derived from macronutrients (*i.e.*, fat, protein and carbohydrates), most mean values were within the DRI levels (Figure 2b) with the exception of the fat % energy intake and carbohydrate % energy intake of Black participants in KwaZulu Natal that were lower than the DRI levels, respectively [19,20]. These results were in contrast to the fat % energy intake of Indian participants in KwaZulu Natal [23], where higher than the DRI levels were observed (Figure 2b). The mean added sugar intakes of all the participants in the reviewed studies seemed to be greater than the 25 g or <10% of energy recommended by the World Health Organization (WHO) [25]. The mean fiber intakes on the other hand were lower in both men and women and were less than that of the RDA of 25 g (W) and 38 g (M) (Table 3), with the exception for the mean fiber intakes of men and women in KwaZulu Natal [19] and the North West [20,21] province, where the intakes were above the recommended amounts (Figure 2c). A notable observation is that the mean energy intakes of rural South Africans appeared to be lower than that of their urban counterparts (Figure 2a) [20,21], with the exception of the results of Tydeman-Edwards [26]. Moreover, despite the observed overall lower mean protein % energy, urban men and women partaking in the Prospective Urban and Rural Epidemiological (PURE) study [20,21] consumed higher percentages (12.6% and 12.5% [20], 13.1% and 13.3% [21], respectively) than their rural counterparts (10.9%, 11.0% [20], 12.1% and 11.9% [21], respectively). In the same studies, the mean percentages total fat intake was lowest in rural participants and the highest in urban ones. 

**Table 1:** Details of the reviewed studies.

Author	Age	Gender	Race	No. of Participants	Area of Study	Urban/Rural	Other Info
Nel and Steyn, 2002 [14]	Adults	Men and Women	Black and White Africans	Adults: Men *n* = 1505 Women: *n* = 1726	South Africa	Both	Secondary data analysis
Tydeman-Edwards, 2012 [26]	Adults (25–64 years)		Mostly Black	Adult men: *n* = 259 Adult women: *n* = 709	Free State	Both	Primary data analysis
Jaffer, 2009 CRIBSA ^‡^ [22]	Adults 25+ years	Men and Women	Black Africans	544	Cape Town	Urban townships (Langa, Gugulethu, Crossroads, Khayelitsha, Nyanga)	Primary data analysis
Hattingh *et al.*, 2008 [27]	25–34 years 35–44 years	Women	Black Africans	496	Bloemfontein	Urban townships (2 formal settlements 2 informal settlements)	Primary data analysis
Oldewage-Theron and Kruger, 2011 [28]	Households	Women and grandmothers	Black (assumption, not mentioned in article)	357	Vaal region—Gauteng province	Peri-urban Informal settlements	Primary data analysis
Msaki and Hendricks, 2013 [29]	Households	Women or other head of household	Black Africans (assumption, not mentioned in article)	200	KwaZulu Natal	Rural community, Embo	Primary data analysis
Msaki and Hendricks, 2014 [30]	Households	Women or other head of household	Black Africans (assumption, not mentioned in article)	200	KwaZulu Natal	Rural community, Embo	Secondary data analysis
Kolahdooz *et al.*, 2013 [19]	Adults	Men and Women	Black Africans (assumption, not mentioned in article)	136	KwaZulu Natal	Rural, Empangeni	Primary data analysis
Audain *et al.*, 2014 [31]	14–21 years	Men and Women	Diverse **	209	KwaZulu Natal	Hilton, peri-urban and rural	Primary data analysis
Labadarios *et al.*, 2011 [32]	16+ years	Men and women	Diverse **	3287	All 9 South African provinces	Urban and rural	Primary data analysis
Shisana *et al.*, 2013 [33]	15+ years	Men and women	Diverse **	13,357	All 9 South African provinces	Urban and rural	Primary data analysis
Naicker, 2009 [23]	Adults (35–55 years)	Men and Women	Indian	Adult men: *n* = 111 Adult women: *n* = 139	KwaZulu Natal	Urban	Primary data analysis
Wentzel-Viljoen and Kruger, 2005 PURE * Data (unpublished) [20]	30–70 years	Men and Women	Black Africans	2009	North West	Urban and rural	Raw data
Wentzel-Viljoen and Kruger, 2010 PURE * Data (unpublished) [21]	30–70 years	Men and Women	Black Africans	1275	North West	Urban and rural	Raw data

* PURE [20,21]: Prospective Urban and Rural Epidemiological study designed to track the changing lifestyles, risk factors and chronic disease among 150,000 people over 15 years across 17 high- to low-income countries from every major developing region in the world. PURE-SA-NWP refers to the South African leg of the PURE study running in the North West Province; ^‡^ CRIBSA [22]: Cardiovascular Risk in Black South Africans study designed to measure the dietary intake of the urban black population of Cape Town twenty years after the BRISK study in 1990. ** Diffent ethnic groups: Black, Coloured, White and Indian/Asian Africans.

**Table 2:** Research methodology used in the reviewed studies.

Author	Aim	Dietary Intake Method	Analysis Method
Naicker, 2009 [23]	To assess the association of dietary and lifestyle exposures with the risk of non-communicable diseases among apparently healthy Indian adults in KwaDukuza, South Africa	Quantitative food frequency questionnaire validated by three quantified 24-h recalls	The quantities of food items recorded were converted to gram weights and the data processed using the South African FoodFinder software Averages of the macro- and micro-nutrients from the three 24-h recalls were compared to the quantities produced by the quantified food frequency questionnaire. Micronutrient intakes were compared with the recommended dietary allowance (RDA) and estimated average intakes (EARs) for all micronutrients
Hattingh *et al.*, 2008 [27]	To assess micronutrient intake of black women living in Mangaung, South Africa	Quantitative food frequency questionnaire (culture sensitive)	The quantities of food items recorded were converted to gram weights and the data processed using the South African FoodFinder software Micronutrient intakes were compared with the recommended dietary allowance (RDA) for all micronutrients except for calcium, chromium, vitamin D, vitamin K, pantothenic acid and biotin where the adequate intake (AI) was used
Jaffer, 2009 CRIBSA ^‡^ [22]	To determine the occurrence of lifestyle risk factors associated with non-communicable diseases. In particular, this specific study focused on the dietary intake and nutritional status of this population in order to ascertain whether dietary patterns/habits have changed in urbanized South Africans since 1990	Quantified 24-h recall	South African FoodFinder software was used to calculate the dietary intake of every person The values of macro and micronutrients were compared with the RDA, the estimated average intakes (EARs) and the acceptable macronutrient distribution ranges (AMDRs)
Oldewage-Theron and Kruger, 2011 [28]	To assess the food security situation of black women in an informal settlement by exploring their food access capabilities through dietary diversity measures and the coping strategies they employ to cope with poverty and hunger	1-week quantified food frequency questionnaire, quantified or 24-h recall and Cornell Hunger Scale	South African FoodFinder software was used to calculate the dietary intake of every person Simple food item count and food group variety scores were calculated to determine dietary diversity scores Nutrient adequacy ratios (NARs) for energy, proteins, carbohydrates and 31 micro-nutrients were calculated by dividing the actual daily intake of nutrients and by the current dietary reference intakes of specific nutrient for women’s age category (IoM) The values of macro and micronutrients were also compared with the EARs
Msaki and Hendricks, 2013 [29]	To understand household food security using food diversity, quality, and intake	Checklist, food item count and screening	Household food intake strata were developed using matrices obtained from the household food intake index and nutritional adequacy ratios Food quality was measured using food count and later using 5 food groups, namely, starches, vegetables and fruits, animal sourced foods, fats, and legumes
Msaki and Hendricks, 2014 [30]	Estimation of micronutrients intake in household food consumption surveys	Household food intake index	The principal component analysis (PCA) involved breaking down household energy, protein and micronutrients per capita intakes (w.r.t. women adult equivalents) into categorical or interval variables The variables were then processed in order to obtain weights and principal component The results obtained from the first component (explaining the most variability) was used to develop the Household Food Intake Index based on formula: *Aj* − *f* 1*x* (*aji*-*a*1)/(*S*1) + ……*fNx* (*fajN*-*aN*)/(*sN*) [34] Using the 33.3 and 66.6 percentile, the resulting household population was divided into three household food intake quintiles representing the inadequate, average adequate and adequate household in terms of food intake
Kolahdooz *et al.*, 2013 [19]	To investigate dietary adequacy amongst adults in rural KwaZulu-Natal, by determining daily energy and nutrient intakes, and identifying the degree of satisfaction of dietary requirements	24-h dietary recall	All dietary data from the interviewer-administered 24-h recalls were coded and analysed using Nutribase version 9 (Cybersoft Inc., Pheonix, AZ, USA), which calculated energy and nutrient intakes per person
Audain *et al.*, 2014 [31]	To make a comparative analysis of the dietary preferences of adolescents attending an urban *versus* a peri-urban school in KwaZulu-Natal, in order to investigate the association between socio-economic status and food frequency	Self-administered non-quantified food frequency questionnaire	Data analysis employed the grouping of food according to groups and assigned the frequency of eating. Responses to consumption frequency were assigned values ranging from 0–8 A score of 0:Never or less than once a month A score of 1: 1–3 times a month A score of 2: once a week A score of 3: 2–4 times a week A score of 4: 5–6 times a week A score of 5: Once a day A score of 6: 2–3 times a day A score of 7: 4–5 times a day A score of 8: 6 or more times a day
Labadarios *et al.*, 2011 [32]	To measure the dietary diversity score (DDS) in South Africans aged 16+ years from all the population groups as a proxy of food insecurity	Face validated 24-h recall which was not quantified	Each specific food item was included in a group of nine selected food groups as used in an earlier study on children. A score below 4 was indicative of poor dietary diversity (and by association poor food security) while a score of nine represented a very varied diet. Each food group was only counted once when calculating DDS. The nine groups used were: (1) cereals/roots/tubers; (2) meat/poultry/fish; (3) dairy; (4) eggs; (5) vitamin A rich fruit and vegetables; (6) legumes; (7) other fruit; (8) other vegetables; (9) fats and oils. The results also included calculating the proportion of people who had consumed a food group at least once
Shisana *et al.*, 2013 [33]	To measure the DDS of South Africans 15+ years by summing the number of food groups from which food had been consumed	24-h recall which was not quantified	The outcome was based on the 9 food groups namely: cereals, roots and tubers; vitamin A-rich vegetables and fruit; vegetables other than vitamin A rich; fruit other than vitamin A-rich fruit; meat, poultry, and fish; eggs; legumes; dairy products; and foods made with fats or oils. A score below 4 was indicative of poor dietary diversity (and by association poor food security) while a score of nine represented a very varied diet. Each food group was only counted once when calculating DDS.
Wentzel-Viljoen and Kruger, 2005 PURE * Data (unpublished) [20]	To determine the occurrence of lifestyle risk factors associated with non-communicable diseases.	Quantified food frequency questionnaire	Macro- and micronutrient intakes were calculated using the South African Medical Research Council (SAMRC) Food Database
Wentzel-Viljoen and Kruger, 2010 PURE * Data (unpublished) [21]	To determine the occurrence of lifestyle risk factors associated with non-communicable diseases. The dietary data in the South African leg of the PURE study focused on the dietary intake and nutritional status of this population in the North West Province in order to ascertain whether dietary patterns/habits have changed in the same participants since 2005 in North West province	Quantified food frequency questionnaire	Macro- and micronutrient intakes were calculated using SAMRC Food Database
Nel and Steyn, 2002 [14]	The primary objective of this study was to generate a reference table of “most commonly” consumed food items and average intakes of these items in the diet of South Africans. The table is required to be representative of foods eaten by children and adults from all age and ethnic groups in South Africa.	Secondary data-analysis was conducted on existing dietary databases (raw data) obtained from surveys undertaken in South Africa between 1983 and 2000.	Data had to be extrapolated from existing isolated surveys on adults. In this process the following databases were utilized: Black Risk Factor Study (BRISK); First Year Women Student (FYWS) Project; Weight and Risk Factor Study (WRFS); the National Food Consumption Survey (NFCS) and the Coronary Risk Factor Study (CORIS). The dietary intake for the groups 1–5 years and 6–9 years were calculated only from the NFCS, and were not supplemented by other databases. The substantiation for treating age 10+ as a unit (and calling it an adult group), was the finding that average consumption of adolescents (10–15 years) did not differ significantly from that of adults when comparing mean energy intakes of age groups in the studies analyzed.
Tydeman-Edwards, 2012 [26]	The main aim of this study was to determine the diet and anthropometric status of adults (between 25 and 64 years old) and pre-school children (zero to seven years old) in rural and urban areas. In addition, this study investigated associations between anthropometric status of children and adults in rural and urban areas in order to determine whether a double burden of disease existed.	A 24-h recall of reported usual intake and adjusted food frequency questionnaire were used to determine dietary intake during individual interviews with each participant.	The exchange lists, based on the American Dietetics Association (ADA) Food Guide Pyramid (United States Department of Agriculture (USDA), 1992: online), classify food into seven groups according to their energy, carbohydrate, fat, and protein content, and these were used to quantify the energy and macronutrient content of the dietary intake of participants. Cut off points were followed such that: food intake less than the recommendations of the Food Guide Pyramid (USDA, 1992: online) were regarded as inadequate or below requirements; intake within the guidelines, as adequate or within requirements; and intake higher than the guidelines, as high or above requirements.

* PURE [20,21]: Prospective Urban and Rural Epidemiological study designed to track the changing lifestyles, risk factors and chronic disease among 150,000 people over 15 years across 17 high- to low-income countries from every major developing region in the world. PURE-SA-NWP refers to the South African leg of the PURE study running in the North West Province; ^‡^ CRIBSA [22]: Cardiovascular Risk in Black South Africans study designed to measure the dietary intake of the urban black population of Cape Town twenty years after the BRISK study in 1990.

**Table 3:** Macronutrient intake of South Africans based on the second dietary analysis of studies undertaken after 2000.

	**Dietary Reference Intakes (DRIs) Food and Nutrition Board [24]**						
	Energy: Men of height 1.70 m of low activity with BMI = 22.5 = 10,626		Fat: AMDR = 20%–35%	Protein: AMDR = 10%–35%	Carbohydrate: AMDR = 45%–65%	Added Sugar ** <10% Energy or 25 g per day Recommended by WHO [25]	Fiber: RDA Males = 38 g
	Energy: Women of height 1.60 m with low activity and BMI = 22.5 = 8465		Fat: AMDR = 20%–35%	Protein: AMDR = 10%–35%	Carbohydrate: AMDR = 45%–65%	<10%E or 25 g per day Recommended by WHO [25]	Fiber RDA Females = 25 g
**Study**	**Gender**	**Energy kJ Mean (SD)**	**Fat % total energy Mean (SD)**	**Protein % total energy Mean (SD)**	**Carbohydrates (CHO) % total energy Mean (SD)**	Added sugar ** (g) Mean (SD)	**Fiber (g) Mean (SD)**
Naicker, 2009 [23]	Men	7815 (1514.1)	35.1 (3.2)	12.8	49.9	59.6 (68.4)	18.8 (4.1)
	Women	7214 (1209.5)	37.1 (3.2)	12.0	47.0	45.4 (46.4)	18.1 (3.8)
Nel and Steyn, 2002 [14]	Men	9788 (5485)	25.1 (12.4)	14.5 (4.5)	59.6 (14.3)	59.6 (68.4)	22 (14)
	Women	7250 (3610)	25.0 (12.2)	14.3 (4.7)	59.9 (14.1)	45.4 (46.4)	18 (12)
Tydeman-Edwards, 2012 [26]	Men (Rural)	8630	25.2	18.3	60.2	na	
	Men (Urban)	7078	23.3	17.5	62.2	na	
	Women (Rural)	7755	25.9	16.9	60.3	na	
	Women (Urban)	6621	22.8	17.7	63.3	na	
Jaffer, 2009 CRIBSA ^‡^ [22]	Men 19–44 years	8600 (3200)	30.1 (12.7)	13.7 (4.8)	53.2 (13.7)	45.0 g (42.8 g)	18.9 (10.4)
	Men 45–64 years	7700 (2200)	25.9 (13.8)	13.4 (5.1)	57.4 (14.1)	49.4 g (37.7 g)	18.1 (10.4)
	Women 19–44 years	7600 (2300)	30.1 (12.7)	12.4 (4.5)	55.5 (12.5)	54.4 g (40.5 g)	16.2 (8.5)
	Women 45–64 years	7100 (1800)	27.6 (14.1)	12.4 (4.9)	57.3 (15.0)	47.0 g (36.3 g)	16.8 (8.2)
Kolahdooz *et al.*, 2013 [19]	Men 19–50 years	11,159	19 (11)	13 (3)	69 (13)	35 g (25 g)	36 (18)
	Men 50+ years	10,874	18 (10)	13 (3)	68 (9)	39 g (53 g)	28 (25)
	Women 19–50 years	11,650	17 (9)	11 (2)	67 (12)	47 g (24 g)	39 (14)
	Women 50+ years	11,978	17 (7)	12 (3)	64 (11)	47 g (21 g)	47 (14)
Wentzel-Viljoen and Kruger, 2005 Unpublished PURE * data [20]	Men (Rural)	6973 (3203)	18.3 (6.3)	10.9 (2.0)	64.2 (9.4)	32 g (28 g)	19 (9)
	Men (Urban)	10,054 (4164)	25.3 (6.9)	12.6 (1.9)	56.5 (6.9)	55 g (33 g)	27 (13)
	Women (Rural)	6107 (2472)	20.3 (7.1)	11.0 (1.7)	66.5 (8.7)	33 g (23 g)	17 (7)
	Women (Urban)	9008 (3899)	28.2 (6.6)	12.5 (2.0)	55.6 (7.0)	58 g (33.5 g)	23 (11)
Wentzel-Viljoen and Kruger, 2010 Unpublished PURE data * [21]	Men (Rural)	10,084 (5709)	23.2 (7.43)	12.1 (3.4)	59.8 (11.3)	62 g (62 g)	27 (19)
	Men (Urban)	15,485 (10,209)	27.2 (7.4)	13.1 (2.4)	54.7 (8.5)	82 g (72 g)	40 (25)
	Women (Rural)	9891 (5528)	24.8 (8.5)	11.9 (3.1)	61.5 (10.5)	66 g (78 g)	27 (19)
	Women (Urban)	12,302 (5876)	27.8 (7.1)	13.3 (2.4)	55.5 (8.5)	81 g (68 g)	33 (16)

BMI = body mass index; AMDR = acceptable macronutrient distribution range; WHO = World Health Organisation; RDA = recommended dietary allowance; SD = standard deviation; na = not available; * PURE [20,21]: Prospective Urban and Rural Epidemiological study designed to track the changing lifestyles, risk factors and chronic disease among 150,000 people over 15 years across 17 high- to low-income countries from every major region of the world. PURE-SA-NWP refers to the South African leg of the PURE study running in the North West Province; ** Added sugars included sugars (sucrose) added by adults or manufacturers. Sugars naturally present in foods such as fructose were not included ^‡^ CRIBSA [22]: Cardiovascular Risk in Black South Africans study designed to measure the dietary intake of the urban black population of Cape Town twenty years after the BRISK study in 1990.

### 3.2. Micronutrient Intakes

Table 4 outlines a summary of mean micronutrient intakes from seven studies namely: PURE [20,21], CRIBSA [22], Hatting *et al.* [27], Kolahdooz *et al.* [19], Oldewage-Theron and Kruger [28] and Naicker [23]. The table presents the minimum mean values and the maximum mean values found among all the studies for each nutrient. As can be noted, the mean calcium and vitamin D intakes of the men and women are far below the recommended amounts (based on DRIs) of 1000 mg and 10 μg, respectively. Based on the maximum mean values found in some studies it appears that iron, zinc, folate, niacin, riboflavin, thiamin and vitamins A, B_6_, B_12_, C and E were above the DRIs in some studies. However, the low mean values, as represented by the minimum means found, indicate that intakes of iron, zinc, folate, niacin, vitamin A and vitamin C were also below the DRIs for certain studies. 

**Table 4:** Summary of micronutrient intake from different studies undertaken after 2000 reporting minimum and maximum values.

Dietary Variable and Their DRIs	Minimum (Lowest) Reported Mean Value out of all 6 Studies	Maximum (Highest) Reported Mean Value out of all 6 Studies
Calcium: AI for M and W = 1000 mg	M = 299 mg [19]; W = 150.5 mg [28]	M = 743.2 mg [21]; W = 636.4 mg [27]
Iron: EAR for M = 6.0 mg, for W = 8.1 mg	M = 8.0 mg [22]; W = 3.8 mg [28]	M = 27.7 mg [21]; W = 29.0 mg [19]
Zinc: EAR for M = 9.4 mg, for W = 6.8 mg	M = 7.6 mg [22]; W = 3.8 mg [28]	M = 21.7 mg [21]; W = 16.6 mg [21]
Folate: EAR for M and W = 320 μg	M = 226 μg [23]; W = 81.9 μg [28]	M = 1633 μg [19]; W = 1763 μg [19]
Niacin: EAR for M = 12 mg, for W = 11 mg	M = 12.8 mg [20]; W = 4.9 mg [28]	M = 38.8 mg [21]; W = 31.8 mg [19]
Riboflavin: EAR for M = 1.1 mg, for W = 0.9 mg	M = 1.0 mg [22]; W = 0.3 mg [28]	M = 2.8 mg [21]; W = 2.4 mg [21]
Thiamin: EAR for M = 1.0 mg, for W = 0.9 mg	M = 0.8 mg [23]; W = 0.7 mg [28]	M = 2.8 mg [19]; W = 3.1 mg [19]
Vitamin A: EAR for M = 625 μg, RE for W = 500 μg	M = 125 μg [19]; W = 196 μg [19]	M = 2159 μg [21]; W = 2132 μg [21]
Vitamin B6: EAR for M and W = 1.1 mg	M = 1.0 mg [21]; W = 0.3 mg [28]	M = 5.3 mg [21]; W = 4.0 mg [21]
Vitamin C: EAR for M = 75 mg, for W = 60 mg	M = 12.6 mg [20]; W = 14.4 mg [28]	M = 90.7 mg [19]; W = 90.1 mg [21]
Vitamin B12: EAR for M and W = 2.0 μg	M = 1.1 μg [19]; W = 1.1 μg [19]	M = 11.2 μg [21]; W = 9.7 μg [21]
Vitamin E: EAR for M and W = 12 mg	M = 8.1 mg [19]; W = 4.6 mg [28]	M = 21.4.1 mg [21]; W = 17.6 mg [21]
Vitamin D: EAR for M and W = 10 μg	M = 2.8 μg [22]; W = 0.7 μg [28]	M = 7.7 μg [19]; W = 9.0 μg [19]

M: men and W: women. Location of studies: North West-PURE 2005, 2010 [20,21]; Bloemfontein [27]; Cape Town CRIBSA [22]; North West-PURE-2010 [21]; Vaal region [28]; Kwa Zulu-Natal [19]; Kwa Zulu-Natal [23]. Dietary reference intakes (DRI), Estimated average requirements (EARs), adequate intakes (AIs), retinol equivalents (RE), recommended dietary allowances (RDAs) and acceptable macronutrient distribution range (AMDR).

### 3.3. Food Intakes

The most commonly consumed food items by South Africans are presented in Table 5. According to the comparison of the three of studies done before 2000 [14] and one undertaken in 2012 [26], the most frequently consumed items were added sugar, tea, maize porridge, brown bread, full cream milk, coffee, white bread, margarine, potatoes, fruit and vegetables and rice. 

**Table 5:** Comparison of 10 most frequently consumed foods by South Africans.

Study on Secondary Analyses (Nel and Steyn [14])	Bloemfontein Men (Tydeman-Edwards [26])	Bloemfontein Women (Tydeman-Edwards [26])
Maize porridge and dishes	Sugar	Sugar
Sugar	Maize porridge	Tea
Tea	Tea	Maize porridge
Brown bread	Stock	Stock/salt
White bread	Coffee	Margarine/oil
Non-dairy creamer	Margarine/oil	Bread
Brick margarine ^1^	Full cream milk	Full cream milk
Chicken meat	Bread	Vegetables
Full cream milk	Vegetables	Fruit
Green leafy vegetables	Fruit	Cold drinks
Potatoes	Cold drinks	Chicken
Tomato and onion stewed	Eggs	Eggs
Coffee	Chicken	Sweets/chocolates
Eggs	Cake/biscuits	Chips
Cabbage	Alcohol	Cakes/biscuits

^1^ Brick margarine—hard (hydrogenated) margarine packaged in a paper cover.

In the CRIBSA study [22], the number of portions consumed daily from different food groups were cereals (M = 8.2; W = 7.3); fat (M = 3.2; W = 4.1); fruit and vegetables (M = 2.2; W = 2.6); followed by the meat (M = 2.1; W = 1.7); and, lastly, the dairy group (M = 0.5; W = 0.4). The number of portions consumed for fruit and vegetables are half of those recommended, *i.e.*, 2.2–2.6 *versus* five recommended a day. And the number of dairy are 0.4–0.5 *versus* at least 2.0 recommended a day.

### 3.4. Dietary Diversity

The two recent national South Africans surveys (Table 6) [32,33] showed that overall mean dietary diversity score (DDS) scores for South Africa are 4.2 and 4.02, respectively. The highest scores are observed in the urban formal settings (4.7 and 4.42, respectively) and the lowest are in the rural and tribal settings (3.3 and 3.17, respectively). Moreover, in both the surveys, Black South Africans appear to have the lowest DDS (4.0 and 3.63, respectively) while White South Africans have the highest DDS (5.6 and 4.96, respectively) [32,33]. 

**Table 6:** Mean dietary diversity scores (DDSs) based on items from nine food groups, according to two South African national studies.

	2012 SANHANES (Shisana *et al.* [33])				2009 Study (Labadarios *et al.* [32])		
	Mean DDS		DDS < 4		Mean DDS		DDS < 4
	Mean	95% CI	Percent	95% CI	Percent	95% CI	
**Area**							
Urban formal	4.7	4.5–4.9	29.3	25.8–33.1	4.42	4.34–4.07	26
Urban informal	3.8	3.5–4.1	46.6	40.7–52.6	3.46	3.30–3.61	55.7
Rural formal	3.6	3.4–3.9	50.7	44.3–57.1	3.64	3.46–3.81	50.1
Rural informal	3.3	3.2–3.5	59.7	54.6–64.7	3.17	3.05–3.29	63.9
**Province**							
Western Cape	4.6	4.3–4.8	28.2	22.5–34.7	4.78	4.66–4.90	15.7
Eastern Cape	4.0	3.7–4.2	42.1	37.1–47.4	3.38	3.22–3.54	59.6
Northern Cape	3.8	3.5–4.1	43.6	35.2–52.5	4.05	3.85–4.26	35.1
Free State	4.0	3.7–4.3	45.1	37.1–53.4	4.40	4.23–4.58	26.6
Kwa-Zulu Natal	3.7	3.5–4.0	49.3	41.9–56.6	3.97	3.81–4.12	40.8
North West	3.3	3.1–3.5	61.3	55.3–67.0	3.72	3.43–4.01	44.1
Gauteng	4.9	4.6–5.2	26.3	21.0–32.2	4.22	4.08–4.31	32.5
Mpumalanga	4.0	3.5–4.4	46.2	37.3–55.4	4.14	3.95–4.33	30.5
Limpopo	3.2	2.8–3.6	65.6	52.8–76.5	4.02	3.03–3.45	61.8
**Race**							
African	4.0	3.8–4.1	44.9	41.1–48.8	3.63	3.55–3.71	50
White	5.6	5.2–6.0	14.9	10.2–21.2	4.96	4.82–5.10	9
Coloured	4.5	4.2–4.7	30.0	26.0–34.4	4.43	4.30–4.56	26
Asian	4.1	3.7–4.6	31.6	20.8–44.9	4.44	4.29–4.58	26
Total SA	4.2	4.1–4.3	39.7	36.7–42.7	4.02	3.96–4.07	38

SANHANES, South African National Health and Nutrition Examination Survey; DDS, dietary diversity score; CI, confidence interval; SA, South Africa.

A local study by Oldewage-Theron and Kruger [28] have shown that households in a peri-urban informal settlements of the Vaal Triangle in the Gauteng province of South Africa presented with a low mean food variety score of 3.17 ± 1.21 and a low mean dietary diversity score of 2.82 ± 0.99 based on 0–6 food groups used in the calculations. 

## 4. Interpreting the Findings

The current review sought to identify dietary studies undertaken in South Africa in an effort to describe diets consumed by adult South Africans and to assess possible dietary deficiencies. Notable findings were that, in total, seven studies provided data on energy and micronutrient intakes. These studies are restricted to the North West urban and rural areas [20,21], Cape Town urban areas [22], Free State urban and rural areas [26], Vaal region [28], and KwaZulu-Natal rural [19] and urban [23] areas. No studies are available from any other provinces.

There are large variations in energy and macronutrient intakes. Energy intakes range from means of 6973 kJ to 15,485 kJ in men and 6107 kJ to 12,302 kJ in women. The mean total energy intake of men and women in South Africa is shown to consistently be lower than the recommendation (except for those living in KwaZulu Natal and the North West province [19,20,21]), which could indicate that a large percentage of men and women take in less than needed. The % energy from protein ranges from 10.9% to 18.3%; fat from 17% to 37.1%; and carbohydrate from 47.0% to 69%. The majority of the mean % values of macronutrients (proteins, fats and carbohydrates) lie within the Acceptable Minimum Distribution Ranges of the DRIs. For instance, the mean % energy from fat is lower than the maximum of 35% in most of the studies while the mean % energy from protein is just above the minimum of 10% in some of these studies which can be considered to be on the low side. The differences in mean values were likely to have been influenced not only by geographic location (urban/rural) but also by dietary methods used. Urban intakes were generally higher than rural intakes. 

It is interesting to note that the main 10 foods consumed are nearly identical in each study. Unfortunately, there are no national data available on portion sizes of foods commonly consumed. The mean DDS for South Africa in 2009 was 4.02 and it increased to 4.2 in 2012. The lowest mean DDS was found in rural informal areas, ranging from 3.17 in 2009 and increasing to 3.33 in 2012. The highest mean DDS was found in urban formal areas increasing from 4.42 in 2009 to 4.70 in 2012. Black South Africans had the lowest mean DDS and White South Africans had the highest in both 2009 and 2012, indicative of poorer food security in Black South Africans [32,33].

South Africa is experiencing rapid urbanization and it is of concern that especially in Kwa-Zulu Natal and the North West Province % energy intake from fat and added sugar of urban Africans are higher than their rural counterparts. Furthermore, intakes of fruit and vegetables are very low in South Africans. The PURE study reported median intake values of less than 150 g for rural and urban men and women [35] in comparison with the recommendation of at least 400 g per day. In addition, the data on the PURE study also indicates a higher intake of micronutrients in the urban than rural South Africans with large percentages of participants not meeting the DRIs [24]. Both too high energy intake especially from fat and sugars and too low micronutrient intakes are contributors to risk for cardiovascular diseases (CVDs) that are alarmingly on the rise in Sub Saharan Africa [36]. The relevance of this study is that this phenomenon is not reported in all the studies, which means that, if correctly addressed, the negative nutrition transition accompanying urbanization can be steered towards a more healthy population. 

The South African government introduced compulsory food fortification in 2005 [37]. However, overconsumption of fortified staple foods (maize porridge and bread), dietary fat, as well as added sugar (not fortified), may be putting South Africans at risk of non-communicable diseases (NCDs), a health crisis that has been highlighted in the country [33,37,38]. For instance, it is evident that some South African communities’ diets (rural and peri-urban) lack food-group diversity, and are very high in cereals (maize), bread and added sugar [32,33,38]. Noteworthy, fruit and vegetables are regarded as good sources of vitamins and minerals and contribute to the fiber intake. However, according to the current review, South Africans are consuming less fruit and vegetables, and in return this could be impacting on the level of micronutrients and fiber in their diet. Evidence regarding the burden of diseases in South Africa, suggests low fruit and vegetable intake to account for 3.2% of total deaths and 1.1% of the 16.2 million attributable to disability-adjusted life years (DALYs) [39]. 

The large consumption of staple foods (maize and bread) may be fueled by the reduced prices linked to these foods since the Government subsidizes these foods, and reimburses large millers and upgrades smaller millers with equipment to reinforce compliance with the fortification legislation [40]. In addition, no Value Added Tax (VAT) is paid on these foods. Hence, in South Africa, staple foods cost less per unit of energy than animal products, fruit and vegetables [41,42], and they are the preferred food choices by most people in poorer communities [32,33]. 

Therefore, within South African food-insecure households, it is common to find women selecting these staple foods when shopping for their family [28,32]. These foods typically contain high quantities of refined starch, with sugar and fat or oil often added to them when they are prepared to enhance flavor and improve their satiety [28,37,43]. It is well-documented that overconsumption of foods that are high in refined cereals, sugar and fat promote weight gain [44]. Of concern is that evidence regarding the burden of diseases in South Africa, suggests that excess weight gain has caused 36,504 deaths (95% uncertainty interval 31,018–38,637) or 7% (95% uncertainty interval 6.0%–7.4%) of all deaths in 2000 [44]. The burden in women was approximately double of that in men. 

Of note is that chicken is the only meat listed in Table 5. Red meat is among the most expensive food items in South Africa [42]; as such it is mainly unaffordable to poorer communities. Thus, chicken appears to be mostly preferred source of protein. Although the studies on foods listed full cream milk as one of the most frequently consumed foods, the calcium intake of the men and women in all the reported studies were below the recommendation implying that the portions consumed were smaller than the recommendations. This could have an impact on the high incidence of hypertension seen in South Africa [45].

Another point of note from our results is that South Africa is a nation that frequently consumes tea and coffee, with the average coffee and tea intakes being about two cups per day [14,26]. Sugar is usually added to these beverages. In addition, other products like sucrose-sweetened beverages also increase the intake of sugar. The high sugar intake in all the studies is confirmed by the studies reporting the most frequently consumed food [14,26]. The recent publication by Vorster *et al.* [38] showed that the proportion of adults who consumed sucrose-sweetened beverages doubled over a five-year period. The mean sugar intake for the urban women was reported to be 147 grams, for those who consumed sugar. As mentioned previously, overconsumption of foods that are high in refined sugar promote weight gain, which in turn is a risk factor for the development of NCDs [44,38]. 

Sucrose-sweetened beverages are often consumed simultaneously with food. This could be impacting on the absorption of some vitamins and minerals, particularly dietary iron. In fact, Morck *et al.* [46] have shown that when a cup of filter or instant coffee is ingested with meals, iron absorption is reduced from 5.88% to 1.64% and 0.97%, respectively. They further highlights that, when the strength of the instant coffee is doubled, the absorption level of iron falls even lower to 0.53%. In effect, both tea and coffee contain chemical compounds called tannins, which are the cause of poor absorption. 

As mentioned before, bread is also one of the preferred staple foods in the country. Based on various South African studies it is calculated that bread contributes between 5% and 35% of sodium intake, depending on the ethnic group being studied [47]. Additionally, in South Africa, salt is added to food when cooking, when eating at the table, and during food processing, and could contribute on average 40% to total sodium intake [45,47]. This is a cause for concern since increased salt intake leads to an increase in blood pressure [45,47]. Hence, the new legislation by the South African government is aimed at salt reduction in bread, breakfast cereals and other products. Furthermore, advocacy for reducing salt intake and its benefits has been intensified [48].

There are some limitations to our study notably the use of different dietary intake methodologies applied in the different studies, the use of a non-South African food composition database in one study [19] and the inconsistency in the method of reporting dietary data. Since the 24-h recall is known to under-report dietary data and the food frequency to over-report data there is a strong possibility that both conditions existed in the various studies examined in this review [49]. The low energy intakes in females in some studies certainly suggest under-reporting, particularly in view of the fact that 65.1% are overweight and obese [32]. Finally, none of the studies reported adjustment for day-to-day variation. The majority of studies compared the mean intakes with the DRIs.

## 5. Conclusions 

There is a paucity of national dietary data on adults in South Africa, with the exception of dietary diversity, which was measured twice in the past few years. However, with the exception of the PURE and CRIBSA studies, there are relatively little representative data on adults. The data which are available indicate that energy intakes are low in certain studies, particularly in rural areas, while adequate to high intakes were found in urban areas. The same apply to fat, protein and carbohydrate intakes, although they generally still remain within Acceptable Minimum Distribution Ranges. However, despite national food fortification, the intake of numerous micronutrients still remain low, particularly calcium, folate, B vitamins, and vitamin C, and D. The studies that showed the lowest mean intakes of micronutrients were Black urban adults in Cape Town [22], Black rural adults in Kwa-Zulu Natal [19], Black women living in informal settlements in the Vaal region [28], and Indian adults in Kwa-Zulu Natal [23]. With regard to the low micronutrient intakes, it is not known whether the monitoring of food fortification is efficient and effective since there has been little research in this regard, or whether intakes are still low despite efficient fortification.

## 6. Recommendations

The importance of national dietary surveys as part of a monitoring and surveillance system is of vital importance to ascertain the nutritional health and wellbeing of the population. Failing this, regular surveys in different provinces can also provide important information to the Department of Health at a lower cost. Ideally such surveys should elicit information on both children and adults and should target poor socio-economic and deep rural areas, and vulnerable groups. Such surveys should use the most reliable and valid dietary assessment methods taking into consideration the health literacy and levels of education of the participants. Ideally, at least a repeated 24 h recall, supported by a food frequency questionnaire may prove to be the best options to use since most studies reported on in this study indicated that they were using validated methods. 

It is further recommended that the effectiveness of the current national food fortification system is evaluated. Are the millers adding the correct levels of fortification mix to the vehicles of fortification, namely wheat flour and maize meal? It is vital that this is checked since the country has a myriad of small millers selling maize meal to the public and it may well be that fortification levels are below those which are legislated.

In addition to monitoring and fortification, the Department of Health and local health authorities should continue to strengthen their health promotion efforts with regard to teaching about a balanced diet for optimal health. In this regard, the recently updated food-based dietary guidelines for South Africans [50] should be used as the basis for educating the population on healthy eating habits to ensure a healthy population. 

Overall, it is recommended that data on representative samples be elicited from other provinces in order to determine macronutrients, micronutrients and commonly eaten foods and their portions sizes. Although programs that are initiated by the South African government (food fortification and regulation of some foods such as salt) are important and show a promise in eradicating micronutrient deficiencies and prevent NCDs, they need to be run effectively if they are to safeguard South Africans’ health. Policy audits need to be done regularly and their enforcement intensified. In essence, the South African government needs to monitor and fast track these policies if they are to see their effectiveness. In the light of the aforementioned evidence, it is also clear that education regarding the importance of moderate total food energy and sugar-added beverage intake is mandatory. Furthermore, the importance of fiber, good fats and sources of proteins as well as vitamin- and mineral-rich fruit and vegetables need to be advocated. The recent updated food-based dietary guidelines for South Africans [50], endorsed by the South African Department of Health, address all these important factors and should be used as the basis for educating the population on healthy eating habits to ensure a healthy population. 
